# Utility of Three-Dimensional Printing for Preoperative Assessment of Children with Extra-Cranial Solid Tumors: A Systematic Review

**DOI:** 10.3390/pediatric14010006

**Published:** 2022-01-11

**Authors:** Sachit Anand, Nellai Krishnan, Prabudh Goel, Anjan Kumar Dhua, Vishesh Jain, Devendra Kumar Yadav, Minu Bajpai

**Affiliations:** 1 Department of Pediatric Surgery, Kokilaben Dhirubhai Ambani Hospital, Mumbai 400053, India; drsachit_anand@outlook.com; 2 Department of Pediatric Surgery, All India Institute of Medical Sciences, New Delhi 110029, India; nellai93@gmail.com (N.K.); anjandhua@hotmail.com (A.K.D.); dr.vishesh79@gmail.com (V.J.); drdevendra@hotmail.com (D.K.Y.); bajpai2b@gmail.com (M.B.)

**Keywords:** three-dimensional printing, extra-cranial, solid tumors, children, pediatric oncology, preoperative planning

## Abstract

Background: In cases with solid tumors, preoperative radiological investigations provide valuable information on the anatomy of the tumor and the adjoining structures, thus helping in operative planning. However, due to a two-dimensional view in these investigations, a detailed spatial relationship is difficult to decipher. In contrast, three-dimensional (3D) printing technology provides a precise topographic view to perform safe surgical resections of these tumors. This systematic review aimed to summarize and analyze current evidence on the utility of 3D printing in pediatric extra-cranial solid tumors. Methods: The present study was registered on PROSPERO—international prospective register of systematic reviews (registration number: CRD42020206022). PubMed, Embase, SCOPUS, and Google Scholar databases were explored with appropriate search criteria to select the relevant studies. Data were extracted to study the bibliographic information of each article, the number of patients in each study, age of the patient(s), type of tumor, organ of involvement, application of 3D printing (surgical planning, training, and/or parental education). The details of 3D printing, such as type of imaging used, software details, printing technique, printing material, and cost were also synthesized. Results: Eight studies were finally included in the systematic review. Three-dimensional printing technology was used in thirty children with Wilms tumor (n = 13), neuroblastoma (n = 7), hepatic tumors (n = 8), retroperitoneal tumor (n = 1), and synovial sarcoma (n = 1). Among the included studies, the technology was utilized for preoperative surgical planning (five studies), improved understanding of the surgical anatomy of solid organs (two studies), and improving the parental understanding of the tumor and its management (one study). Computed tomography and magnetic resonance imaging were either performed alone or in combination for radiological evaluation in these children. Different types of printers and printing materials were used in the included studies. The cost of the 3D printed models and time involved (range 10 h to 4–5 days) were reported by two studies each. Conclusions: 3D printed models can be of great assistance to pediatric surgeons in understanding the spatial relationships of tumors with the adjacent anatomic structures. They also facilitate the understanding of families, improving doctor–patient communication.

## 1. Introduction

Safe surgical resection of the tumor is one of the fundamental principles in the management of solid tumors in children. High-quality imaging modalities including computed tomography (CT) and/or magnetic resonance imaging (MRI) form the basis of diagnostic imaging and assessment of resectability in children with suspected malignant solid tumors [1]. However, both these investigations provide a two-dimensional view of the tumor and the adjoining structures. Three-dimensional (3D) reconstruction and visualization, using special software, do provide a better spatial relationship, but in the majority of these cases, they are also viewed from a two-dimensional screen or panel [2]. Thus, a precise topographic view is lacking in these scenarios. In comparison, the use of 3D printed models provides a 360-degree view of the tumor and the adjoining structures. They have been shown to be superior to conventional radiological investigations for preoperative surgical planning [3,4]. Apart from a better visualization, they also provide a sense of touch to the surgeons for better comprehension of the surgical anatomy. Various studies highlighting the usage of 3D printing technology for oncological resections in adults have been conducted [5]. However, the published literature for children with extracranial solid tumors is limited. This systematic review was conducted to evaluate the role of 3D printing as a preoperative tool for children with extracranial solid tumors. 

## 2. Materials and Methods

### 2.1. Search Process

The present study was registered on PROSPERO (registration number: CRD42020206022) [6]. The search process was conducted according to the Preferred Reporting Items for Systematic Reviews and Meta-Analyses (PRISMA) statement [7]. The PubMed database was explored independently by two authors (PG and SA) to exclude a prior systematic review on the same topic. Subsequently, three authors (SA, PG and NK) systematically searched PubMed, Embase, SCOPUS, and Google Scholar on 15 September 2021. The search terms included: Term 1-“Three dimensional printing” OR “Three-D printing” OR “3-D printing” OR “3-dimensional printing” **AND** Term 2-“pediatric tumor” OR “pediatric surgical oncology” OR “pediatric extracranial solid tumor” OR “pediatric solid tumor” OR “Wilms tumor” OR “Neuroblastoma” OR “Hepatoblastoma” OR “Gem cell tumor” OR “retroperitoneal tumor”. Once identified, the duplicates were removed and the relevant studies were screened based on the eligibility criteria.

### 2.2. Inclusion/Exclusion Criteria

**Figure 1:**
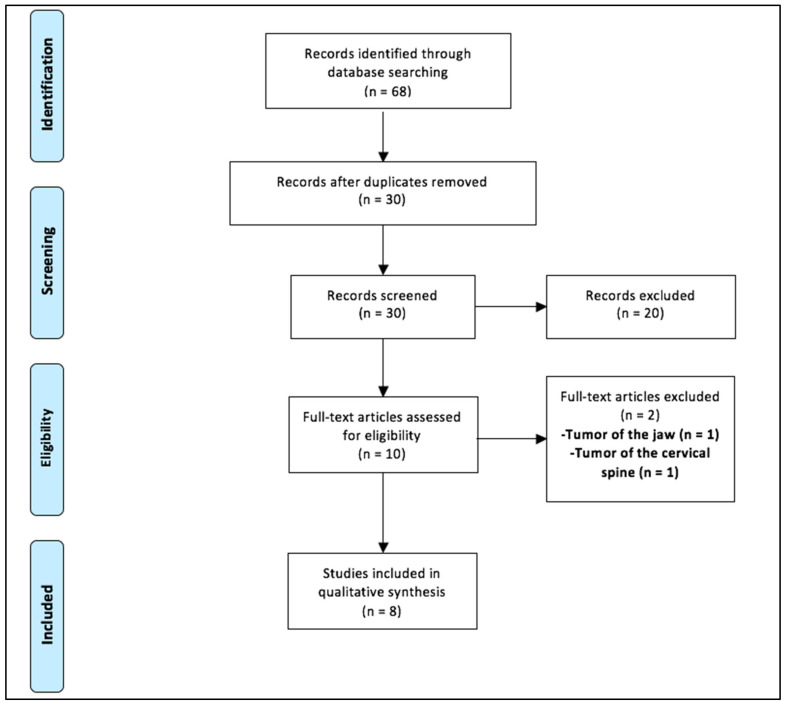
Selection of the relevant studies using the Preferred Reporting Items for Systematic Review and Meta-Analysis (PRISMA) flow diagram.

The inclusion criteria were based on: Participants-studies including patients up to 18 years of age and harboring extracranial solid tumors; Intervention: usage of three-dimensional printed tumor models; Controls: no controls; Outcome: the utility of 3D printing in preoperative assessment, training/education, and counseling of parents or guardians; Study type: All study designs, i.e., randomized controlled trials (RCTs), cohort studies, case-control studies, case series, and individual case reports were eligible for inclusion. Comment and opinion pieces, review articles, editorials, letters to the editor, and abstracts were excluded. Additionally, children with orthopedic tumors, tumors of the jaw, and tumors involving the spine were excluded during the full-text review. Studies published in languages other than English were also excluded. A detailed PRISMA flow diagram of the search strategy is included in Figure 1. 

### 2.3. Data Extraction

After the inclusion of the relevant studies, data were extracted by two observers (AD and VJ) independently. Data on each study, including the author(s), study design, year of publication, number of patients, age and gender of patients, details of the tumors (including the type of tumor and the organ of involvement), and the planned surgical procedure were recorded. Additionally, information on the type of imaging used for 3D reconstruction, details of the software used for data acquisition and analysis, the technique and type of material used for 3D printing, the number of printed models per study, and cost of each model were extracted in an extraction table using MS Excel software (Version 15.24). Any discrepancies among the investigators were resolved by a senior author (MB). 

### 2.4. Quality Assessment

Methodological quality assessment of the included case reports and case series was performed by utilizing a tool proposed by Murad et al. [8]. A slight modification of this tool in this present study was to exclude questions 4, 5 and 6 from the causality domain because these were specific for adverse drug events. After the quality assessment was done by two observers (PG and DKY) independently, the measurement of the inter-observer agreement was done using kappa statistics. Based on the kappa values, the level of agreement was defined as almost perfect (0.81–1.00), substantial (0.61–0.80), moderate (0.41–0.60), fair (0.21–0.40), slight (0.00–0.20) and poor (<0.00) [9].

## 3. Results

### 3.1. Study Characteristics

A total of 68 articles were identified using the search strategy (Figure 1). After the removal of the duplications, 30 abstracts were screened. Of these, 20 were excluded, and only 10 studies were eligible for full-text review. Two of these were further excluded, as these focused on juvenile ossifying fibroma of the jaw and tumor involving the cervical spine [10,11]. Finally, eight were included in the systematic review (Table 1). Of these, three were cross-sectional/survey studies, three were case series, and two were case reports. 3D printing technology was used for a total of thirty children with extracranial solid tumors. Out of these, thirteen (43.3%) had Wilms tumor and seven (23.3%) had neuroblastoma. Hepatoblastoma, retroperitoneal tumor, and synovial sarcoma were present in one case each. Although Yang et al. included seven children with hepatic tumors who were planned for hepatectomy, there was no mention of the specific types of tumor [12]. The study and patient characteristics are depicted in Table 1.

Of the eight included studies, five employed the technology of 3D printing for preoperative surgical planning [3,5,13,14,15]. Additionally, an improved understanding of the surgical anatomy of solid organs was depicted in two of these studies [2,4]. Yang et al. stressed the application of 3D printing for improving the parental understanding of the tumor and its management [12]. 

### 3.2. Details of Preoperative Imaging and 3D Printing Process

Computed tomography and magnetic resonance imaging were either performed alone or in combination for radiological evaluation in these children (Table 2). The scans were loaded as digital imaging and communications in medicine (DICOM) files. Subsequently, segmentation of the anatomic structures of interest and 3D reconstruction was performed by special pieces of software (Table 2). These files were saved as stereolithography (.STL) files suitable for printing into a 3D model. The different types of printers and printing materials used in the included studies are listed in Table 2. Two studies utilized different materials to depict vascular anatomy and renal parenchyma (including tumor) in their printed models [5,13]. The cost of the 3D printed models was explicitly reported by two studies only. The expenses were around 450–500 USD per model [4,12]. Similarly, the manufacturing time was reported by two studies only. It ranged from 10 h to 4–5 days [4,13]. 

### 3.3. Methodological Quality Assessment

The detailed quality assessment scoring by two independent observers for each study is depicted in Table 3. The domains of ascertainment and reporting were adequately addressed by the included studies. However, the weaknesses included the selection and causality domains. Kappa statistics showed a value of 0.769 (*p* < 0.001), highlighting a substantial agreement among the two observers.

## 4. Discussion

The technology of 3D printing was first introduced by Charles Hull in 1984 with the creation of the first stereolithography machine [16]. Over the years, this technology has gained immense popularity among the various surgical specialties. Its rapid growth in the fields of orthopedics and maxillofacial surgery has been remarkable. This is probably because the 3D printing process involving the bony structures and its interpretation are less complex than those for the soft tissues [17].

The first report illustrating the role of 3D printing in pediatric malignancies was published by Souzaki et al. in 2015 [15]. The present systematic review provides various applications of 3D printing for children with extracranial solid tumors. Of all the potential roles of 3D printing, a better understanding of the surgical anatomy and subsequent surgical planning is noteworthy [2,3,4,5,13,14,15]. Possible reasons for improved identification of the anatomic structures with 3D models have been put forward by Yang et al. [2]. It is believed that the anatomic representation is simpler with 3D models as compared to conventional imaging (multi-detector computed tomography), as the latter requires comprehensive knowledge of radiology. Additionally, these models provide a sense of touch to the surgeons, thus offering a better comprehension of the surgical anatomy. Finally, the printed models are portable objects, and can be sterilized and taken to the operating room for intraoperative assistance [2,13]

Sánchez-Sánchez A et al. [3] have demonstrated their experience with 3D printed models in complex pediatric solid tumor resections. In all four cases in this study, 3D reconstruction and printing were instrumental in providing a better spatial relationship of the structures for easy manipulation and tumor resectability. The printed model was shown to be immensely helpful in the first case (with bilateral Wilms tumor), where it provided precise evidence of sufficient normal renal parenchyma for the feasibility of nephron-sparing surgery (NSS). Óscar Girón-Vallejo et al. also highlighted the usefulness of a 3D printed model for planning NSS in a ten-month-old infant with bilateral Wilms tumor. Other advantages with regards to these children include anticipation of complications like renal atrophy, urinary fistula, etc. [5]. 

Souzaki et al. [15] showed the application of 3D printing for preoperative planning for a three-year-old child with PRETEXT IV hepatoblastoma. The liver model was helpful in understanding the surgical anatomy of the mass located at the porta hepatis. Based on the surgical simulation, the child underwent extended left hepatectomy. It is believed that 3D printed models can be extremely useful in this subset of patients. It helps us to precisely identify the children who will benefit from resection, therefore restricting the option of liver transplantation to unresectable tumors only. The technology can also aid in identifying any unusual anatomic variations of the liver during the exercise of preoperative planning [3].

Krauel et al. [13] demonstrated the use of physical 3D models in preoperative planning for three tumors encasing major vessels. It was highlighted that 3D printing can help in achieving greater and safer resections in tumors such as neuroblastoma and synovial sarcoma. A similar experience was shared by Sánchez-Sánchez A et al. [3] when discussing two cases of neuroblastoma. Another application of 3D printing in neuroblastoma was shown by Souzaki et al., where 3D models were not only used to plan the sites of port insertion but also to simulate the steps of laparoscopic adrenalectomy [14]. 

Apart from usage in surgical planning, 3D printed models can be a great source of teaching and medical education. Relations of the various anatomic structures can be explained in detail using these models [2]. In a survey study among seven pediatric surgeons, the conventional imaging (CT and or MRI) of ten patients with Wilms tumor was compared with 3D models and augmented reality (AR) holograms. It was found that the evaluation of all anatomic structures (tumors, arteries, veins, and urinary collecting structures) was significantly better with the 3D models and AR holograms as compared to conventional imaging. However, no significant difference was observed between 3D printing and the AR holograms [4]. 

Another important role of the use of the 3D printed model for these children was highlighted by Yang et al. [12] in their study. 3D models proved to be an excellent source of parental education. It was demonstrated that there is a significant improvement in parental knowledge and understanding of the liver anatomy, liver physiology, tumor characteristics, planned surgical procedure, and risks of the surgery. In the present era, it is extremely important to educate parents about the basic details of the tumor and the available surgical options along with their risks, and in the majority of these interactions, the parents are non-medical persons. Therefore, surgeons can provide all the necessary information to parents with the help of printed models, and this exercise can improve the doctor–patient relationship.

Although 3D printed models have an additional value for children with extracranial solid tumors, there are some limitations associated with them. The main limiting factor of this technology is its availability. Due to its cost, the facility of 3D printing is selectively available. On average, one model costs 450–500 USD [4,12] Additionally, the procedure of 3D printing is time-consuming, taking 10 h to a few days [4,13] Secondly, there are some technical limitations associated with the simulations, such as the reproducibility of the actual tissue characteristics in printed models. This problem is particularly encountered while resecting the tumors and dividing the vessels due to their rigid consistencies. Additionally, these models fail to express real-time scenarios such as post-chemotherapy adhesions, bleeding, etc., [13,14]. Finally, high-quality preoperative imaging (CT, MRI, or both) is required for precise 3D printed models [4]. Thus, the entire procedure, starting from image acquisition to model printing, needs to be standardized in the future.

The present study has a focused research question. The reason for not including intracranial tumors and for excluding orthopedic tumors (and tumors of the jaw) was based on the forte and expertise of General Pediatric Surgeons. This systematic review paves the way for further research, and it will be interesting to study the utility of 3D printing for these abovementioned and unexplored tumors. 

## 5. Conclusions

3D printing is an extremely useful modality for children with extra-cranial solid tumors. In comparison to conventional imaging modalities, a topographic view and sense of touch are the characteristic advantages of this technology. It can help in planning complex oncological resections precisely, provides an insight into the anatomic variations in organs (e.g., the liver), and helps in organ-preserving surgeries (nephron-sparing surgery). It is a valuable tool for teaching and educating residents and medical students. Finally, it is a great modality to improve parental understanding about the tumor characteristics and planned surgical procedure, along with the risks of the surgery. Given the available studies (only case reports and case series), the level of evidence of our findings is limited. 

## Figures and Tables

**Table 1:** Characteristics of the included studies.

S N	Author/Year	Study Design	n	Type of Tumor
1	Wellens LM et al./2019	Cross sectional	10	Wilms tumor (3-bilateral)
2	Sánchez A, et al./2018	Case series	4	Wilms tumor ^#^ (n = 2); Neuroblastoma (n = 2)
3	Yang T, et al./2018	Cross sectional	7	Hepatic tumors
4	Yang T, et al./2018	Cross sectional	1	Retroperitoneal tumor
5	Vallejo OG, et al./2017	Case report	1	Bilateral Wilms tumor
6	Krauel L, et al./2015	Case series	3	Neuroblastoma (n = 2); Synovial sarcoma (n = 1)
7	Souzaki R, et al./2015	Case series	3 *	Neuroblastoma (n = 3)
8	Souzaki R, et al./2015	Case report	1	Hepatoblastoma

* One printed model was used for three cases of adrenal neuroblastoma. ^#^ One with bilateral pulmonary metastasis.

**Table 2:** Details of imaging and 3D printing.

S N	Author/Year	Preoperative Imaging	3D Segmentation Software	3D Printing Technology	Material Used
1	Wellens LM et al./2019	MRI (n = 10); CT (n = 3)	Mimics Innovation Suite	3D printing technology (Z corporation) at Materialise	Composite material
2	Sánchez A, et al./2018	MRI (n = 3); CT (n = 1)	Cella-supplied (Cella Medical Solutions, Spain)	Fused deposit modelling (FDM) and injection printing at BCN technologies	Plastic-derived materials like polylactic acid, acrylonitrile butadiene styrene (ABS), polyvinyl, etc.
3	Yang T, et al./2018	CT	Mimics software	RS6000 rapid prototyping printer (Shanghai Union 3D technology corp.)	Photosensitive resin
4	Yang T, et al./2018	CT	Mimics software	RS6000 rapid prototyping printer (Shanghai Union 3D technology corp.)	Photosensitive resin
5	Vallejo OG, et al./2017	MRI	Cella-supplied (Cella Medical) Solutions, Spain	A combination of material injectors with 3D printers (Cella Medical Solutions)	Polylactic acid-vessels; transparent polyurethane rubber for renal parenchyma and tumor
6	Krauel L, et al./2015	CT and MRI	VRMed DICOM platform	-Polyjet 3D printing using Connex 5000 by Stratasys -SLS 3D model made in Vanguard machine	Epoxy photopolymer-bones, vessels; soft translucent material-tumor
7	Souzaki R, et al./2015	CT	3D workstation (ZedView, 3D Doctor, FreeForm and CATIA)	Objet 500 connex 3 device (Stratasys)	Acrylic ultraviolet curable resin
8	Souzaki R, et al./2015	CT	3D workstation (ZedView, 3D Doctor, FreeForm and CATIA)	Objet 500 connex 3 device (Stratasys)	Acrylic ultraviolet curable resin

Abbreviations: MRI, Magnetic resonance imaging. CT, Computed tomography. 3D, Three-dimensional. DICOM, Digital imaging and communications in medicine.

**Table 3:** Methodological quality assessment by two independent observers.

Assessment by Observer 1						
S N	Author/Year	Domains				
		Selection	Ascertainment		Causality	Reporting
1	Wellens LM et al./2019	1	1	1	N/A	1
2	Sánchez A, et al./2018	1	1	1	0	1
3	Yang T, et al./2018	1	1	1	N/A	1
4	Yang T, et al./2018	1	1	1	N/A	1
5	Vallejo OG, et al./2017	0	1	1	0	1
6	Krauel L, et al./2015	1	1	1	0	1
7	Souzaki R, et al./2015	1	1	1	1	1
8	Souzaki R, et al./2015	0	1	1	1	1
**Assessment by Observer 2**						
S **N**	**Author/Year**	**Domains**				
		**Selection**	**Ascertainment**		**Causality**	**Reporting**
1	Wellens LM et al./2019	1	1	1	N/A	1
2	Sánchez A, et al./2018	1	1	1	0	1
3	Yang T, et al./2018	1	1	1	N/A	1
4	Yang T, et al./2018	1	1	1	N/A	1
5	Vallejo OG, et al./2017	0	1	1	1	1
6	Krauel L, et al./2015	1	1	1	0	1
7	Souzaki R, et al./2015	1	1	1	0	1
8	Souzaki R, et al./2015	0	1	1	1	1

## Data Availability

The data presented in this study are available upon request of the respective author.
